# Can moderate dietary salt restriction help patients with hypertension?

**Published:** 2023-04

**MARCH 22, 2023 -** Results from a clinical trial published in the Journal of Internal Medicine reveal several health benefits of moderate salt restriction in patients on standard medical treatment for primary aldosteronism.

Primary aldosteronism—a condition in which the adrenal glands make too much of the hormone aldosterone—is a common cause of secondary hypertension. The combination of aldosterone excess and high dietary salt intake leaves affected patients with a higher risk of cardiovascular disease than patients with hypertension from other causes. Mineralocorticoid antagonists are the main treatment of primary aldosteronism, but these medications do not completely normalize patients’ elevated cardiovascular risk.

Because elevated aldosterone and high dietary salt intake have detrimental effects on patients’ health, investigators wondered whether salt restriction might benefit patients. In the non-randomized single-arm Salt CONNtrol trial that included 41 patients, moderate salt restriction reduced blood pressure and depressive symptoms without detectable adverse effects.

“The study shows that a moderate dietary salt restriction is feasible, when combined with a dedicated smartphone app for continuous motivation, and has a strong antihypertensive effect in patients with primary aldosteronism,” said corresponding author Christian Adolf, MD, of Ludwig Maximilian University of Munich, in Germany. “Our findings will help to improve care for patients with primary aldosteronism and, likely, also for subgroups of patients with essential hypertension.”

***Link to Study: https://onlinelibrary.wiley.com/doi/10.1111/joim.13618***

***Full citation:** “Moderate dietary salt restriction improves blood pressure and mental well-being in patients with primary aldosteronism: The salt CONNtrol trial.” Holger Schneider, Anna-Lina Sarkis, Lisa Sturm, Vera Britz, Andreas Lechner, Anne L. Potzel, Lisa Marie Müller, Daniel A. Heinrich, Heike Künzel, Hanna F. Nowotny, Thomas Marchant Seiter, Sonja Kunz, Martin Bidlingmaier, Martin Reincke, Christian Adolf. J Intern Med; Published Online: 22 March 2023 (DOI: 10.1111/joim.13618).*

*Copyright © 2019 The Cochrane Collaboration. Published by John Wiley & Sons, Ltd., reproduced with permission.*

